# Clinical Features of Inflammatory Myopathies and Their Association with Malignancy: A Systematic Review in Asian Population

**DOI:** 10.1155/2013/509354

**Published:** 2013-02-25

**Authors:** Patompong Ungprasert, Napat Leeaphorn, Nattamol Hosiriluck, Wikrom Chaiwatcharayut, Nischala Ammannagari, Donald A. Raddatz

**Affiliations:** ^1^Department of Internal Medicine, Bassett Medical Center and Columbia University College of Physicians and Surgeons, Cooperstown, NY 13326, USA; ^2^Department of Internal Medicine, Phramongkutklao College of Medicine, Bangkok 10440, Thailand; ^3^Department of Internal Medicine, Faculty of Medicine, Chulalongkorn University, Bangkok 10330, Thailand

## Abstract

*Introduction*. Idiopathic inflammatory myopathies (IIMs) are a group of chronic systemic autoimmune diseases that mainly affect the skeletal muscle. The common subtypes include adult dermatomyositis (DM), polymyositis (PM), and inclusion body myositis (IBM). Most of the earlier studies that described the clinical characteristics of IIM as well as their association with cancer were conducted in Western population. Our study is the first systematic review that summarizes the clinical data of DM/PM in Asian population. *Methods*. We identified 14 case series of DM/PM that met our eligibility criteria. We then compared this data with that from previous reports from Europe and North America. *Results*. Our systematic review included 2518 patients. Dermatomyositis is more common, with the ratio of dermatomyositis to polymyositis being 1.36 : 1. 69% of them were females with mean age of 45.5 years. Extramuscular manifestations, including arthritis/arthralgia, dysphagia, and interstitial lung disease, are found in one-third of the patients. Malignancy was found in 10% of patients, with lung and nasopharyngeal carcinomas being the most common malignancies associated with these myopathies. *Conclusion*. Clinical presentation of PM/DM appears to be similar in both Western and Asian populations. However, the type of associated malignancies in Asians differs from that in Caucasians. Ethnic background should be one of the factors that clinicians should consider while screening for malignancy.

## 1. Introduction

Idiopathic inflammatory myopathies (IIMs) are a group of chronic systemic autoimmune diseases that mainly affect the skeletal muscle. The common subtypes include adult dermatomyositis (DM), polymyositis (PM), and inclusion body myositis (IBM). In 1975, Bohan and Peter proposed the diagnostic criteria for DM and PM using clinical, laboratory, and pathological features which remain to be the gold standard for use in clinical studies [1, 2]. Malignancies associated with these myopathies have been extensively reported in the medical literature since 1916 and then confirmed by subsequent metaanalyses [3–5].

Most of the earlier epidemiological studies that describe the clinical characteristics of IIM as well as their association with cancer come from Western population [4–6]. However, in recent years, several case series have been reported in the Asian population. This study aims to report a systematic review of case series reported exclusively in Asia in order to better characterize the demographics, the clinical data, and the associated malignancies in patients with DM/PM in this population.

## 2. Material and Methodology

We identified published case series that reported the patients with DM and PM that met the Bohan and Peter criteria for DM/PM (definite, probable, and possible) (Table 1) by searching the PubMed database (until November 2012) [1, 2]. We used the search terms “*Polymyositis*” OR “*Dermatomyositis*” and restricted our review to human study. We also manually searched the reference lists of all initially selected articles. We included only studies that were done in Asian countries in an attempt to summarize the clinical data of DM/PM exclusively in this population. When more than one case series was reported from the same institution or used the same database, we included only the case series with the largest number of patients and that with the most detailed clinical data to avoid duplication. We excluded case series that reported on fewer than five patients to minimize potential bias of reporting nonrepresentative cases. 

## 3. Data Assessment

Our search strategy yielded 71 potentially relevant articles. After full length article review, 6 of them were excluded due to potential patient duplication [7–12], one study was excluded since it included only patients with cancer [13], one study was excluded since it did not use the Bohan and Peter criteria [14], and another study was excluded since it was a nonconsecutive case series [15].

**Figure 1:**
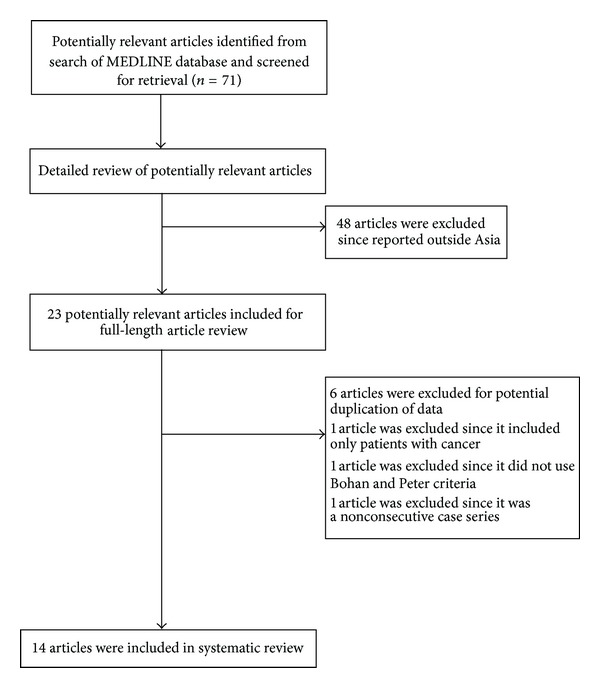
Case series identification.

14 case series met our inclusion criteria and were included for data analysis [16–29].  Figure 1 outlines our search methodology and selection process.

## 4. Results

Tables 2 and 3 summarize the clinical and laboratory data along with the incidence of associated malignancy of the 14 case series that met our inclusion criteria. These case series comprised a total of 2518 patients. The largest studies contributed to more than a half of all the patients [25]. 7 studies were done in East Asia [21–25, 27, 28], 3 studies were done in South East Asia [17, 19, 29], 2 studies were conducted in South Asia [16, 20], and the other 2 studies were from the Middle East [18, 26]. 

### 4.1. Demographics and Clinical Features

Female predominance is seen in all series, ranging from 57% to 81%. Patients tend to present in their fifth to sixth decade with the mean age being 45.5 ± 5.1 years. Dermatomyositis is more common in these series with the ratio of dermatomyositis to polymyositis of 1.36 : 1. Extramuscular manifestations include arthritis/arthralgia (35.0 ± 10.2%), dysphagia (29.3 ± 13.2%), and interstitial lung disease (37.5 ± 18.3%).

### 4.2. Laboratory Findings

Creatine kinase is elevated in the majority of patients with a mean of 3019.2 ± 1088.4 IU/L. Positive antinuclear antibody (ANA) is found in nearly half of the patients (53.7 ± 10.1%), while Anti-Jo-1 antibody is found in 10.7 ± 3.8%.

### 4.3. Association with Malignancy

Malignancy is found in 10.0 ± 6.4% of these patients. It is more prevalent in patients with DM than those with PM (12.3 ± 8.5% and 5.5 ± 7.8%, resp.). Nasopharyngeal carcinoma and lung cancer are the most common types of cancer (1.88%), followed by breast, colon, gastric, and hepatobiliary tract cancers (1.14%, 0.79%, 0.79%, and 0.79%, resp.).

## 5. Discussion

Most of the earlier epidemiological studies characterizing the clinical presentation of IIM come from Western countries, especially from Northern Europe, leaving the clinical manifestations of these diseases in Asian population unclear. However, in the recent years, several case series from Eastern population have been described in the literature. Here, we report the first systematic review of PM/DM in Asian population, emphasizing the clinical and laboratory features as well as their association with malignancy.

Consistent with the Caucasian studies, there is a striking female predominance, mostly in the fifth to the sixth decade of life (female to male ratio is 2.2 : 1). However, there are a couple of relatively small case series from India and the Middle East that reported an average age of mid-thirties. In contrast to the PM predominance in European and North American cohorts [30–34], DM is more prevalent in our study population, with the DM to PM ratio of 1.36 : 1 [25]. This might signify the role of ultraviolet radiation in their pathogenesis, as previously described in a study by Okada et al. [35].

Symmetric, progressive proximal muscle weakness is the hallmark of these myopathies. However, several nonmuscular manifestations have been commonly described. Our cohort is noted to have dysphagia in approximately one-third, which is consistent with the other epidemiological studies from Europe and North America [33, 36–38]. Interstitial lung disease (ILD), one of the major causes of morbidity and mortality in patients with IIM, is also found in about one-third of our patients, although the incidence varies widely from 11% to 74%. This variability is, at least, partly related to the different methods used to identify ILD in each study and is also noted in studies from Western countries [39, 40]. 

ANA is consistently positive in approximately a half of the patients included in our systematic review. Anti-Jo-1 antibody, the most common type of myositis-specific autoantibody, is found in about 10% of our patients which is lower than 18–20% from 3 large studies from Europe and USA [41–43]. However, this finding is not unexpected, since ethnic variation in the frequency of myositis specific autoantibodies has been described. For example, anti-Jo-1 antibody is found in only 3% and 0% in Meso-American (Mexico and Guatemala) and French-Canadian populations, respectively [44, 45]. 

High incidence of malignancy is, again, noted in our study (12.3 ± 8.5% and 5.5 ± 7.8% for DM and PM, resp.). Our incidence is comparable with data from Caucasian population [30–32, 34]. However, the frequency of a specific type of associated cancer in our population is different when compared to Caucasian studies. Nasopharyngeal carcinoma, which is rarely found in Caucasian cohort, is the most common type of cancer in this study (along with lung cancer), while gastric and hepatobiliary tract cancers, which are also uncommonly described in Caucasian studies, are the third most common ones. Interestingly, ovarian cancer, the predominant associated cancer in European and North American cohort, is relatively uncommon in our study, with the incidence of 0.43% (the 8th most common associated cancer). These findings might confirm the role of IIM as a paraneoplastic process that can occur with any malignancy, and the particular type of malignancy associated may vary in different ethnic populations. Thus, ethnic background should be one of the factors that clinicians should consider while screening for malignancy. For example, based on our systematic review, attention should be particularly paid towards Ear-Nose-Throat exam while screening for cancer in DM/PM patients of Asian descent.

## 6. Conclusion

In conclusion, this is a systematic review of PM/DM conducted in Asian population, as an attempt to better characterize the clinical characteristics and the incidence of associated malignancies in this population. Clinical presentation of PM/DM appears to be similar in both Western and Asian countries. Interestingly, even though with comparable overall incidence of malignancy, the incidence of a specific type of malignancy differs from the Caucasian population. These findings might ascertain the role of IIM as a paraneoplastic process, and ethnic background should be one of the factors that clinicians should consider while screening for malignancy.

## Figures and Tables

**Table 1:** Bohan and Peter diagnostic criteria for dermatomyositis/polymyositis.

(1) Symmetrical weakness of limb-girdle muscles and anterior neck flexor	
(2) Muscle biopsy shows evidence of characteristic myositis	
(3) Elevated serum skeletal muscle specific enzyme, especially creatinine phosphokinase	
(4) Electromyographic (EMG) evidence of myositis	
(5) Typical dermatomyositis rash, including heliotrope and Gottron's papules	

Definite polymyositis: all of items (1)–(4); probable: any three of items (1)–(4); possible: any two of items (1)–(4).

Definite dermatomyositis: item 5 plus any three of items (1)–(4); probable: item 5 plus any two of items (1)–(4); possible: item 5 plus any one of items (1)–(4).

**Table 2:** Demographic and clinical characteristics of consecutive patients diagnosed with PM/DM in Asia.

	Prasad et al. [16]	Koh et al. [17]	Maoz et al. [18]	Louthrenoo et al. [19]	Porkodi et al. [20]	Wakata et al. [21]	Kang et al. [22]	Lee et al. [23]	Tani et al. [24]	Chen et al. [25]	Mustafa and Dahbour [26]	Azuma et al. [27]	So et al. [28]	Teh et al. [29]
Demographic data														
Country	India	Singapore	Israel	Thailand	India	Japan	Korea	Korea	Japan	Taiwan	Jordan	Japan	Korea	Malaysia
Total number	66	75	35	100	51	92	66	41	23	1655	30	121	151	12
Number of PM (%)	38 (58)	35 (47)	15 (43)	72 (72)	24 (47)	64 (70)	22 (33)	25 (61)	9 (39)	643 (39)	11 (37)	51 (42)	53 (35)	3 (25)
Number of DM (%)	28 (42)	40 (53)	20 (57)	28 (28)	27 (53)	28 (30)	44 (67)	16 (39)	14 (61)	1012 (61)	19 (63)	70 (58)	98 (65)	9 (75)
Women, %	61	65	57	78	70	68	81	68	74	69	63	71	61	58
Mean age, years	33.0	50.3	53.0	45.0	34.0	60.0	43.7	43.7	54.0	44.3	34.3	54.3	49.5	57.8

Clinical features														
Dysphagia, %	45	11	NA	49	NA	NA	15	NA	NA	NA	40	23	26	33
ILD, %	NA	14	11	23	NA	NA	40	NA	74	NA	48	54	33	33
Arthralgia/arthritis, %	41	34	NA	20	NA	NA	38	NA	NA	NA	20	46	28	NA
Overlaps with other connective tissue disease, %	14	20	6	45	NA	NA	NA	NA	13	NA	10	24	NA	NA

Laboratory features														
Positive ANA, %	NA	47	NA	69	NA	NA	64	NA	NA	NA	60	49	46	42
Positive anti-Jo-1 Ab, %	NA	NA	NA	NA	NA	NA	14	NA	13	NA	7	14	7	8
Mean CK, IU/l	NA	NA	NA	2058	NA	NA	3778	2732	NA	NA	3633	1864	4230	2717

Associated malignancy														
Total, n (%)	2 (3)	17 (23)	13 (37)	5 (5)	1 (2)	12 (13)	6 (8)	16 (27)	3 (13)	128 (8)	0 (0)	20 (17)	25 (17)	5 (42)
PM, n (%)	0 (0)	2 (6)	4 (27)	1 (1)	0 (0)	2 (3)	NA	6 (24)	0 (0)	33 (4)	0 (0)	3 (6)	2 (4)	0 (0)
DM, n (%)	2 (7)	15 (38)	9 (45)	4 (14)	1 (4)	10 (36)	NA	5 (31)	3 (21)	95 (9)	0 (0)	17 (24)	23 (24)	5 (56)

**Table 3:** Overall and individual study incidence of specific types of cancer.

Type of malignancy, *n* (%)	Prasad et al. [16]	Koh et al. [17]	Maoz et al. [18]	Louthrenoo et al. [19]	Porkodi et al. [20]	Wakata et al. [21]	Kang et al. [22]	Lee et al. [23]	Tani et al. [24]	Chen et al. [25]	Mustafa and Dahbour [26]	Azuma et al. [27]	So et al. [28]	Teh et al. [29]	Total (incidence, %)
Country	India	Singapore	Israel	Thailand	India	Japan	Korea	Korea	Japan	Taiwan	Jordan	Japan	Korea	Malaysia	
Nasopharynx	NA	3 (4.0)	0 (0)	NA	NA	1 (1.1)	NA	1 (2.4)	1 (4.3)	32 (1.9)	0 (0)	0 (0)	2 (1.3)	3 (25)	43 (1.88)
Lung	NA	4 (5.3)	1 (2.8)	NA	NA	2 (2.2)	NA	1 (2.4)	0 (0)	27 (1.6)	0 (0)	0 (0)	8 (5.2)	0 (0)	43 (1.88)
Breast	NA	1 (1.3)	2 (5.7)	NA	NA	0 (0)	NA	3 (7.2)	1 (4.3)	13 (0.7)	0 (0)	1 (0.8)	4 (2.6)	1 (8)	26 (1.14)
Colon	NA	3 (4.0)	0 (0)	NA	NA	0 (0)	NA	0 (0)	0 (0)	10 (0.6)	0 (0)	3 (2.5)	1 (0.7)	1 (8)	18 (0.79)
Gastric	NA	0 (0)	0 (0)	NA	NA	2 (2.2)	NA	1 (2.4)	1 (4.3)	1 (0.1)	0 (0)	8 (6.6)	5 (3.3)	0 (0)	18 (0.79)
Hepatobiliary	NA	2 (2.6)	0 (0)	NA	NA	4 (4.4)	NA	1 (2.4)	0 (0)	9 (0.5)	0 (0)	0 (0)	2 (1.3)	0 (0)	18 (0.79)
Lymphoma/leukemia	NA	0 (0)	4 (11.4)	NA	NA	0 (0)	NA	0 (0)	0 (0)	5 (0.3)	0 (0)	1 (0.8)	1 (0.7)	0 (0)	11 (0.48)
Ovary	NA	2 (2.6)	1 (2.8)	NA	NA	1 (1.1)	NA	0 (0)	0 (0)	2 (0.1)	0 (0)	3 (2.5)	1 (0.7)	0 (0)	10 (0.43)
Bladder	NA	0 (0)	1 (2.8)	NA	NA	1 (1.1)	NA	1 (2.4)	0 (0)	4 (0.2)	0 (0)	0 (0)	0 (0)	0 (0)	7 (0.31)
Thyroid	NA	0 (0)	0 (0)	NA	NA	0 (0)	NA	1 (2.4)	0 (0)	3 (0.2)	0 (0)	0 (0)	2 (1.3)	0 (0)	6 (0.26)
Pancreas	NA	0 (0)	0 (0)	NA	NA	1 (1.1)	NA	0 (0)	0 (0)	2 (0.1)	0 (0)	2 (1.7)	1 (0.7)	0 (0)	6 (0.26)
Esophagus	NA	1 (1.3)	1 (2.8)	NA	NA	0 (0)	NA	0 (0)	0 (0)	1 (0.1)	0 (0)	0 (0)	0 (0)	0 (0)	3 (0.13)
Other	NA	1 (1.3)	3 (8.5)	NA	NA	4 (4.5)	NA	2 (4.8)	0 (0)	19 (1.1)	0 (0)	2 (1.7)	0 (0)	0 (0)	31 (1.36)
